# Course of intellectual functioning in schizophrenia and bipolar disorder: a 10-year follow-up study

**DOI:** 10.1017/S0033291721004645

**Published:** 2023-04

**Authors:** Camilla Bärthel Flaaten, Ingrid Melle, Erlend Gardsjord, Thomas Bjella, Magnus Johan Engen, Anja Vaskinn, Gina Åsbø, Kristin Fjelnseth Wold, Line Widing, Siv Hege Lyngstad, Beathe Haatveit, Carmen Simonsen, Torill Ueland

**Affiliations:** 1 NORMENT, Division of Mental Health and Addiction, Oslo University Hospital & Institute of Clinical Medicine, University of Oslo, Oslo, Norway; 2 Department of Psychology, University of Oslo, Oslo, Norway; 3 Division of Mental Health and Addiction, Unit for Early Intervention in Psychosis, Oslo University Hospital, Oslo, Norway; 4 Division of Mental Health and Addiction, Nydalen DPS, Oslo University Hospital, Oslo, Norway; 5 Centre for Research and Education in Forensic Psychiatry, Oslo University Hospital, Oslo, Norway; 6 Early Intervention in Psychosis Advisory Unit for South East Norway, Division of Mental Health and Addiction, Oslo University Hospital, Oslo, Norway

**Keywords:** Schizophrenia, bipolar disorder, intellectual functioning, cognitive development, longitudinal

## Abstract

**Background:**

Intellectual functioning (IQ) is lower in schizophrenia patients compared to healthy controls, with bipolar patients intermediate between the two. Declines in IQ mark the onset of schizophrenia, while stability is generally found post-onset. There are to date few studies on long-term IQ development in bipolar disorder. This study presents 10-year follow-up data on IQ, including premorbid IQ estimates, to track the developmental course from pre-onset levels to long-term outcomes in both patient groups compared to healthy controls.

**Methods:**

We included 139 participants with schizophrenia, 76 with bipolar disorder and 125 healthy controls. Mixed model analyses were used to estimate developmental slopes for IQ scores from estimated premorbid level (NART IQ) through baseline (WASI IQ) measured within 12 months post-onset, to 10-year follow-up (WASI IQ), with pairwise group comparisons. The best fit was found using a model with a breakpoint at baseline assessment.

**Results:**

Only the schizophrenia group had significant declines from estimated premorbid to baseline IQ levels compared to controls. When comparing patient groups, schizophrenia patients had steeper declines than the bipolar group. Increases in IQ were found in all groups over the follow-up period.

**Conclusions:**

Trajectories of IQ from premorbid level to 10-year follow-up indicated declines from estimated premorbid level to illness onset in both patient groups, followed by increases during the follow-up period. Schizophrenia patients had a steeper decline than bipolar patients. During follow-up, increases indicate developmental improvement for both patient groups, but with a maintained lag compared to healthy controls due to lower premorbid levels.

## Introduction

Intellectual functioning (IQ) encompasses the ability to reason and adaptively solve problems in everyday life (Wechsler, 1944). In consonance with this, cognitive function and IQ have been found to be important predictors of functional outcome in both schizophrenia (Bowie & Harvey, 2006) and bipolar disorder (Baune & Malhi, 2015; Mora, Portella, Forcada, Vieta, & Mur, 2013). Furthermore, IQ provides a backdrop for successful use of other cognitive functions, as evidenced by more severe and widespread impairment in schizophrenia patients with lower IQ (Weickert et al., 2000). IQ is also associated with symptom severity and clinical outcome (Leeson et al., 2011), and may indicate rehabilitation potential (Amoretti et al., 2018). Together these observations underline the importance of increasing our knowledge about IQ development in schizophrenia and bipolar disorder as it could provide insight into the mechanisms of these disorders. To date, few studies have followed premorbid to long-term IQ-trajectories. The present study seeks to model IQ development from premorbid levels to long-term follow-up in schizophrenia and bipolar disorder, compared to healthy controls.

Lower premorbid IQ is well-documented in schizophrenia with several studies showing developmental delays (Bora, 2014a; Meier et al., 2014; Woodberry, Giuliano, & Seidman, 2008). Recruitment and registry studies have documented further decline near the onset of illness (Caspi et al., 2003; Sørensen et al., 2010). Similarly, a meta-analysis by Trotta, Murray, and MacCabe (2015) found premorbid impairment in both disorders when assessed retrospectively, although no significant premorbid impairment was found for bipolar disorder when IQ was assessed prospectively. Further, declines near the onset of bipolar disorder are not indicated by recruitment data (Zammit et al., 2004). On the contrary, the risk for bipolar disorder is mainly associated with higher premorbid IQ (Smith et al., 2015). Genetic risk studies mirror the abovementioned findings, showing that risk for schizophrenia is associated with lower IQ, while both higher and lower functioning is associated with risk for bipolar disorder (Smeland et al., 2019). In brief, while the literature on premorbid IQ in schizophrenia consistently indicates deficits, the evidence is less clear for bipolar disorder.

Regarding the development of IQ following the onset of illness, findings from longitudinal studies of schizophrenia mainly indicate stability on a group level (Kahn, 2019). Some studies, however, have found decrements over time relative to controls, a finding which has been interpreted as an illness-specific decline (Zanelli et al., 2019), or alternatively a reflection of a lack of practice effects (Hedman, van Haren, van Baal, Kahn, & Hulshoff Pol, 2013). Furthermore, a few studies have identified clinical and cognitive subgroups showing signs of IQ decline (Barder et al., 2015; Leeson et al., 2011). Possible reasons for this heterogeneity in post-onset development, include illness-related processes, medication effects and loss of participation in education and work.

So far, the literature in bipolar disorder has found little evidence of deteriorated IQ after illness-onset (Burdick, Goldberg, & Harrow, 2010; Hinrichs et al., 2017; Mur, Portella, Martinez-Aran, Pifarre, & Vieta, 2008; Samamé, Martino, & Strejilevich, 2014). Yet, due to some evidence of cognitive decline associated with number of episodes and duration of illness, a hypothesis of neurodegeneration has been put forward (Cardoso, Bauer, Meyer, Kapczinski, & Soares, 2015; Goodwin, Martinez-Aran, Glahn, & Vieta, 2008). Factors potentially contributing to neurodegenerative effects, such as inflammatory processes and neuroanatomical changes, have been identified in bipolar disorder (Buoli, Serati, Caldiroli, Cremaschi, & Carlo Altamura, 2017). Despite this line of evidence, findings related to cognitive course are inconclusive. To date, few studies have investigated the long-term intellectual course in bipolar disorders that include healthy control comparison groups. This, in addition to low correspondence between cross-sectional and longitudinal studies, make conclusions regarding the cognitive trajectory in bipolar disorder difficult to draw (Van Rheenen et al., 2020).

In the general population, IQ increases moderately from young adulthood into middle age, with evidence of continued development of vocabulary into old age (Hartshorne & Germine, 2015). Verbal and visual reasoning remain stable until old age, while basic cognitive processes decline during late adulthood (Hülür, Ram, Willis, Schaie, & Gerstorf, 2016). Notably, durable increases in IQ are expected due to education (Ritchie & Tucker-Drob, 2018). Standardized IQ scores are generally stable over time, given that norms take expected development into account. Thus, they might not reflect trajectories of actual performance (Panayiotou et al., 2020).

The main aim of the current study was thus to investigate the course of IQ, from estimated premorbid levels through baseline assessment, conducted within the first 12 months post-onset, to a 10-year follow-up, in patients with schizophrenia, bipolar disorder, and in healthy controls. Based on previous studies we expected the schizophrenia group to perform below controls on the premorbid estimate, while our hypothesis for the bipolar group was more open based on mixed premorbid findings in this group. At baseline, lower IQ in both patient groups was expected, with the bipolar group performing intermediately between schizophrenia-spectrum patients and controls. We also expected declines from premorbid to baseline IQ in both patient groups, as well as stability over time in both patient groups and controls during the follow-up period.

## Methods

### Participants

Participants were recruited as part of the Thematically Organized Psychosis (TOP)-sample at the Norwegian Center for Mental Disorder Research (NORMENT). The patient sample was recruited from hospitals and outpatient clinics in the larger Oslo area between 2005 and 2012, with a 10-year follow-up assessment running consecutively from 2015. Baseline assessments were done within 12 months after the first treatment for psychosis or mania. Using statistical records, control participants were randomly selected from the same catchment area. Baseline- and most follow-up assessments were carried out at NORMENT, located at Oslo University Hospital, in a laboratory setting with designated rooms for interviews and testing. Participants who were not able to travel for follow-up assessments were assessed at their local outpatient clinics.

We included 139 participants with schizophrenia-spectrum disorders (74.1% schizophrenia, 11.5% schizoaffective disorder and 14.4% schizophreniform disorder), 76 participants with bipolar disorder (bipolar type 1) and 125 healthy controls. All had completed baseline clinical and cognitive assessments and were eligible for participation in follow-up assessments. Of these, 40 in the schizophrenia group, 34 in the bipolar group and 91 healthy controls completed IQ assessments at 10-year follow-up. The retention rate for participants returning that had IQ data at the time of the study was 38.31%. for the patient sample. Healthy controls were screened, excluding individuals with a history of drug abuse in the last 12 months, severe mental illness, or close family members with severe mental illness. Exclusion criteria for both groups were clinically significant head injury, IQ < 70 and inability to complete testing in Norwegian. Written informed consent was obtained from all participants, and the study is approved by the Regional Committee for Medical Research Ethics and the Norwegian Data Inspectorate.

### Clinical measures

Diagnoses were determined using the DSM-IV criteria, based on the Structured Clinical Interview for DSM-IV axis 1 (SCID-I; First, Spitzer, Gibbon, & Williams, 1995) and available information from medical records. Assessments were conducted by trained clinical psychologists and medical doctors, supervised by senior scientists. Symptom severity was measured at both time points using the Global Assessment of Functioning, split version, Symptom scale (GAF-S; Pedersen, Hagtvet, & Karterud, 2007).

### IQ measures

Neuropsychological assessments were administered by psychologists (for the clinical sample) or psychology students trained in standardized testing (for healthy controls), all calibrated on the test battery and supervised by a neuropsychologist. Current IQ was measured using the four-subtest version of the Wechsler Abbreviated Scale of Intelligence (WASI). These scores are age-corrected. Premorbid intelligence was measured with the NART, which consists of 50 orthographically irregular words to be read out loud. The Norwegian NART was recently re-validated in a large sample of individuals with schizophrenia and bipolar disorder (Vaskinn et al., 2020). Standardized scores are calculated from raw error scores correcting for age (Vaskinn et al., 2020). Both measures were administered at baseline and 10-year follow-up. To confirm the reliability of our premorbid measure we investigated the stability of the NART over 10 years in each group. The NART is not well-suited for dyslexic individuals. Thus, analyses were done both excluding and including participants with dyslexia. Analyses excluding participants with dyslexia, are reported in Online Supplementary Tables S10–S12.

### Measures of functioning

The level of functioning at baseline and follow-up was assessed using the Global Assessment of Functioning, split version, Functioning scale (GAF-F; Pedersen et al., 2007).

### Statistical analyses

All statistical analyses were performed using the Statistical Package for the Social Sciences (SPSS) for Windows version 26. For testing group differences on demographic variables and clinical characteristics, ANOVAs with post-hoc Bonferroni corrected comparisons were used for continuous variables and chi-square analyses were used for categorical variables. Repeated measures ANOVAs with Bonferroni corrected post-hoc comparisons were used to investigate change at the subtest/raw score level.

Prior to choosing mixed models we divided the sample into low (below 85 IQ) and high (above 100 IQ) performers and ran repeated-measures ANOVAs to investigate differences in WASI change over time depending on performance level. We found no difference in the change over time depending on baseline IQ and, despite a baseline difference between completers and non-completers in the schizophrenia group, proceeded with using the entire sample in mixed model analyses.

To estimate test-retest reliability before using the NART scores in further analyses, the intraclass correlation (ICC) (Haggard, 1958), with single measures as type and absolute agreement as model, was calculated in each group using a two-way mixed model (Koo & Li, 2016). Mean differences between WASI IQ from baseline to follow-up was investigated using paired-sample *t* tests.

Next, multilevel analyses using the SPSS linear mixed model function were performed on the IQ scores from premorbid estimates, baseline, and 10-year follow-up assessment, using data from all participants. Initially, three different growth curves with fixed and random effects of time on IQ were made separately for each group, starting with a model estimating time as linear (see Online Supplementary Table S7), then with a quadratic effect of time (see Online Supplementary Table S8) and lastly using a breakpoint at baseline and separately estimating linear effects of time from premorbid measure to baseline (T1) and from baseline to follow up (T2) (see Online Supplementary Table S9). Comparing model fit using Akaikes information criteria (AIC), the latter was found to have the best fit for all groups (Online Supplementary Tables S7–S9). This model was run to do comparisons between the groups. A model comparing all three groups had a higher AIC (5868.204) than pairwise comparisons (HC *v.* SZ: 4580.192; HC *v.* BD: 3713.036; BD *v.* SZ: 3426.299), with the latter also having the advantage of fewer factors/covariates, and thus higher statistical power. For this reason, pairwise comparisons were used, but with stricter significance criteria to correct for multiple comparisons.

In the initial growth curves, time was coded as 0 (premorbid), 2 (baseline) and 12 (follow-up) to approximately reflect the intervals between estimated premorbid measures, baseline measures and 10-year follow-up measures of IQ. Accordingly, in our model, premorbid IQ was entered as estimated 2 years before illness onset as this interval is representative for the time between age at onset and baseline assessment in most patients. For the group comparisons with the breakpoint, time was coded as two separate variables (Time1: 0, 2, 2 and Time2: 0, 0, 10). The final model can be described by the formula:
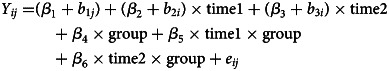
where *Y_ij_* is IQ for person *i* = 1…340 at year *j* = 0…12, *β* signifies fixed effects, *b* signifies random effects, and e is the error term. Group comparisons were made separately for the diagnostic groups, coding healthy controls as 0 and patient groups as 1. An autoregressive heterogeneous covariance matrix was used, and estimates were based on maximum likelihood.

To explore potential moderation by clinical state, we calculated bivariate correlations between GAF-scores and NART IQ, as well as WASI IQ at baseline and follow-up. The alpha level was set at 0.05 for all analyses. Potential medication effects were investigated with *t* tests in each patient group to test for IQ differences between users and non-users, followed by correlations between dosage and IQ.

## Results

Demographic and clinical characteristics of the groups at baseline and follow-up are shown in Table 1. The schizophrenia group exhibited higher levels of symptoms and lower levels of functioning than the bipolar group at both time points, while both groups showed improvements in symptoms and functioning at follow-up. Premorbid, baseline and follow-up IQ measures are shown in Table 2. As expected, the schizophrenia group had lower scores than healthy controls, with the bipolar group intermediate between them. Repeated-measures ANOVAs indicated significant group differences in raw scores for each subtest. Only the Vocabulary subtest changed significantly over time (*F*(1) = 50.72, *p* < 0.000). No group by time interactions were found.

**Table 1.** Demographics and clinical characteristics

M (s.d.)	Schizophrenia *N* = 139	Bipolar *N* = 76	Healthy control *N* = 125	Post hoc and χ^2^ comparisons
Demographics:				
Age	27.29 (8.47)	31.04 (10.55)	30.26 (7.35)	SZ < HC | SZ < BD
Gender: male % (*N*)	61.20 (85)	40.80 (31)	52.80 (66)	SZ > HC | SZ > BD
Years of education	11.94 (2.92)	13.60 (2.31)	14.23 (2.21)	SZ < HC | SZ < BD
Time to follow-up: M (range)	10.20 (9–12)	10.36 (9-13)	9.32 (8-12)	SZ > HC | BD > HC
Clinical measures:				
GAF-S baseline	39.25 (10.16)	56.79 (12.45)		SZ < BD
GAF-S follow-up	54.78 (16.16)	66.32 (13.81)		SZ < BD
GAF-F baseline	41.58 (10.52)	51.62 (12.14)		SZ < BD
GAF-F follow-up	55.35 (15.48)	68.88 (17.92)		SZ < BD
Duration of illness	3.66 (5.18)	3.66 (6.39)		

SZ: schizophrenia group; BD: bipolar group; HC: healthy controls; GAF-F: Global Assessment of Functioning, functioning score; GAF-S: Global Assessment of Functioning, symptom score.

**Table 2.** IQ measures

M (s.d.)	Schizophrenia	Bipolar	Healthy control	Pairwise comparisons
NART, premorbid IQ^1^	108.46 (7.15)	110.71 (7.19)	113.31 (6.01)	SZ < BD < HC
WASI baseline IQ	98.87 (14.29)	107.88 (11.77)	114.09 (9.56)	SZ < BD < HC
WASI follow-up IQ	109.70 (14.29)	113.76 (11.52)	117.82 (8.96)	SZ < HC | BD < HC
Block design:				
Baseline:	46.49 (13.78)	48.28 (14.69)	56.93 (9.76)	HC > BD > SZ
Follow-up:	47.73 (15.37)	48.50 (13.80)	56.96 (9.77)	HC > SZ | HC > BD
Vocabulary:				
Baseline:	57.50 (10.26)	58.97 (7.18)	62.02 (5.97)	HC > SZ | BD > SZ
Follow-up:	60.69 (11.00)	63.23 (8.01)	64.91 (5.76)	HC > SZ
Matrix reasoning:				
Baseline:	26.20 (4.55)	27.56 (4.32)	29.45 (2.82)	HC > BD > SZ
Follow-up	27.18 (4.13)	27.72 (4.52)	29.63 (2.64)	HC > SZ | HC > BD
Similarities				
Baseline:	36.44 (5.38)	37.71 (5.18)	39.59 (4.06)	HC > BD > SZ
Follow-up:	37.24 (5.21)	38.13 (4.06)	39.76 (4.40)	HC > SZ

SZ: schizophrenia group; BD: bipolar group; HC: healthy controls; NART: National Adult Reading Test; WASI: Wechsler Abbreviated Scale of Intelligence.

1 Measured at baseline.

The ICC for NART was then calculated for each group. In the schizophrenia group, the single measure ICC was 0.876 with a confidence interval from 0.763 to 0.937 (*F*(31, 31)) = 15,41, *p* < 0.000. ICC in the bipolar group was 0.885 with a confidence interval from 0.758 to 0.948 (*F*(24, 24)) = 16.01, *p* < 0.000. For healthy controls, ICC was 0.882 with a 95% confidence interval from 0.827 to 0.921 (*F*(90, 90)) = 16.20, *p* < 0.000. Overall, excellent reliability of the NART was found for each group (Fleiss, Levin, & Paik, 2013).

Paired-samples *t* tests for the whole sample showed a significant difference between baseline and follow-up scores for WASI IQ, (*t* = −6.38, *p*(156) < 0.000) but not for NART IQ (*t* = 0.68, *p*(147) = 0.498). The same overall effects were found when analyses were split by groups (See Online Supplementary Table S6). While WASI IQ changed significantly from baseline to follow-up, both ICC and *t* tests indicate a high degree of stability in the NART. Having established this, baseline NART scores were then entered into the multilevel model as premorbid IQ.

**Fig. 1.**
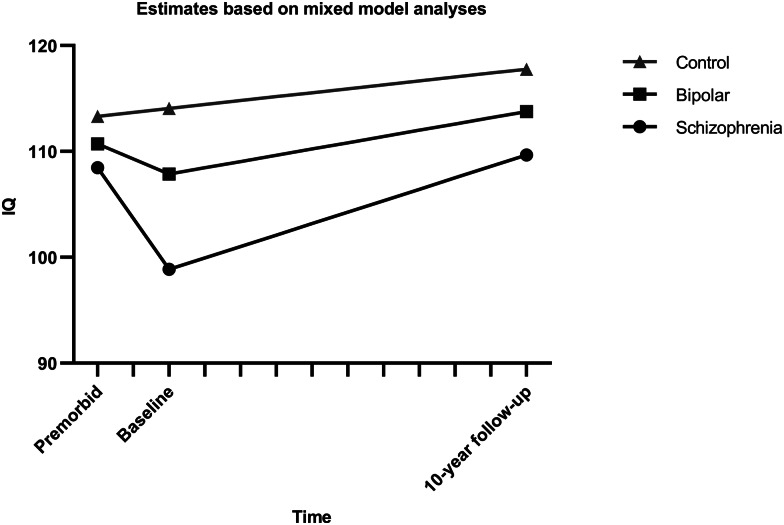
Estimated IQ trajectories from multilevel models with pairwise group comparisons. Intercepts and slopes modeled on estimates from mixed model analysis of the course of IQ, using a break-point model with separate slopes for the interval from premorbid estimate to baseline and from baseline to follow-up.

Based on the model fit of the initial growth models, multilevel models with pairwise comparisons of the groups were done with separate time variables for the periods premorbid-baseline (T1) and baseline-follow-up (T2) (see Online Supplementary Tables S7–S9). Figure 1 shows the estimated effect for the group comparison for all pairwise comparisons. Analyses excluding participants with dyslexia showed the same pattern of effects (Online Supplementary Tables S10–S12).

Model parameters underlying the comparison of the schizophrenia group and healthy controls are found in Table 3. Significant interactions between group and both time-intervals were found, with an estimated decrease in IQ score of 9.6 points (4.80 points per year) for schizophrenia patients during T1 and increases for the schizophrenia group during T2 of 10.8 points (1.08 points per year), while healthy controls increased by 0.76 points during T1 and 3,7 points during T2. Common effects across groups were as expected, indicating increases over time during follow-up and higher scores in healthy controls compared to patients.

**Table 3.** Model parameters for comparison between schizophrenia and healthy controls

Parameter	Estimate	SE	*T*	sig.	95% CI	
					Lower	Upper
Intercept (SZ)	108.46	1.02	105.98	0.000	106.45	110.47
Group (HC)	4.85	1.37	3.59	0.000	2.17	7.53
Time 1	−4.80	0.68	−7.10	0.000	−6.12	−3.47
Time 2	1.08	0.18	5.92	0.000	0.72	1.44
Time 1 × group (HC)	5.18	0.93	5.56	0.000	3.35	7.01
Time 2 × group (HC)	−0.71	0.23	−3.08	0.002	−1.16	−0.26

SZ: schizophrenia group; HC: healthy controls; Intercept: estimated mean premorbid IQ for the schizophrenia group; Group: effect of healthy controls; Time 1: estimated increase per year from premorbid to baseline; Time 2: estimated increase per year from baseline to follow-up; Time 1  × group: interaction effect of Time 1 for the healthy controls; Time 2  × group: interaction effect of Time 2 for the healthy controls.

Model parameters of the comparison of the bipolar group and controls are shown in Table 4. There was a trend-level effect of the group, indicating a 2.5 point higher IQ score at a premorbid level for healthy controls. During T1, a drop of about 2.84 points (1.42 per year) was estimated for the bipolar group compared to 0.78 points for healthy controls, but again the effects were only trend-level. The only effect reaching significance, was a modeled increase of 5.9 points (0.59 points per year) during follow-up, while the estimated lower rate of increase (−2.1) for healthy controls than the bipolar group did not reach significance. All in all, bipolar patients did not differ significantly from controls, or significantly decrease in IQ scores from premorbid to baseline levels, but both groups increased significantly from baseline to follow-up.

**Table 4.** Model parameters for comparison between bipolar and healthy controls

Parameter	Estimate	SE	*T*	sig.	95% CI	
					Lower	Upper
Intercept (BD)	110.71	1.10	101.11	0.000	108.56	112.86
Group (HC)	2.50	1.35	1.92	0.055	−0.06	5.26
Time 1	−1.42	0.76	−1.88	0.061	−2.90	0.07
Time 2	0.59	0.19	3.15	0.002	0.22	0.95
Time 1 × group (HC)	1.81	0.94	1.92	0.056	−0.05	3.66
Time 2 × group (HC)	−0.21	0.22	−0.96	0.337	−0.65	0.22

BD: bipolar group; HC: healthy controls; Intercept: estimated premorbid IQ for the bipolar group; Group: effect of healthy controls; Time 1: estimated increase per year from premorbid to baseline; Time 2: estimated increase per year from baseline to follow-up; Time 1  × group: interaction effect of Time 1 for the healthy controls; Time 2  × group: interaction effect of Time 2 for the healthy controls.

Model parameters underlying the comparison between the two patient groups are described in Table 5. The groups did not differ significantly at the intercept, but both groups had a significant decrease during T1 and an increase during T2. A significant interaction between group and T1 was found with the bipolar group showing a smaller decrease (−2.84 points) from premorbid to baseline levels than the schizophrenia group (−9.6 points). The model indicates the same increase during follow-up of about 10.8 IQ points (1.08 per year), although the non-significant interaction between group and time would mean an increase of only 5.9 points for the bipolar group.

**Table 5.** Model parameters for comparison between patient groups

Parameter	Estimate	SE	*t*	sig.	95% CI	
					Lower	Upper
Intercept (SZ)	108.46	1.15	94.33	0.000	106.20	110.72
Group (BD)	2.25	1.81	1.24	0.215	−1.31	5.81
Time 1	−4.80	0.76	−6.32	0.000	−6.29	−3.30
Time 2	1.08	0.21	5.27	0.000	6.79	1.49
Time 1 × group (BD)	3.38	1.23	2.75	0.006	0.96	5.79
Time 2 × group (BD)	−0.49	0.31	−1.57	0.117	−1.11	0.12

SZ: schizophrenia group; BD: bipolar group; Intercept: estimated premorbid IQ for the schizophrenia group; Group: effect of bipolar group; Time 1: estimated increase per year from premorbid to baseline; Time 2: estimated increase per year from baseline to follow-up; Time 1  × group: interaction effect of Time 1 for the bipolar group; Time 2  × group: interaction effect of Time 2 for the bipolar group.

To evaluate the potential modifying effects of symptoms, we investigated the associations between the GAF-S at baseline and measures of IQ. We found a weak but statistically significant correlation between GAF-S and both NART and WASI IQ at baseline (*r* = 0.250, *p* = 0.043 and *r* = 0.268, *p* = 0.022 respectively), but not between GAF-S and WASI IQ at follow-up for the bipolar group. No significant correlations were found for the schizophrenia group, or for any associations with the GAF-F.

Comparison of medication use *v.* non-use at baseline and follow-up found no significant effects for either patient group. Correlations between medication dose (defined daily dose of lithium, antipsychotics and antiepileptics) at baseline and follow-up found no significant correlations in the schizophrenia group. In the bipolar group, we found negative correlations between IQ and both antipsychotic (WASI IQ: *r* = −.34, *p* = 0.003, NART IQ: − 0.31, *p* = 0.010) and antiepileptic (WASI IQ: *r* = −0.28, *p* = 0.02) dosage at baseline, but no significant association with lithium.

## Discussion

Our main finding is differences in IQ development from premorbid levels (NART) to illness onset (WASI-IQ at baseline), and from illness onset to 10-year follow-up (WASI-IQ at follow-up) in patients with schizophrenia and with bipolar disorder. This is best described as two separate slopes with IQ declines from the premorbid level to illness onset, followed by increases during the long-term follow-up period. Group by time interactions for the premorbid to baseline period indicated larger decline in schizophrenia than in bipolar disorder, while the decrease in the bipolar group only reached significance in the comparison of the patient groups. During follow-up the schizophrenia group increased more than controls, while the rate of increase in the bipolar group was not different from controls. As expected, schizophrenia patients had lower IQ scores than controls, whereas the bipolar group was intermediate between the two, although not significantly different from either.

Our premorbid IQ-measure showed adequate test-retest reliability. The high stability of NART IQ estimates over a 10-year interval validates its use as a premorbid measure. Our results expand on those of other studies finding long-term stability (Morrison, Sharkey, Allardyce, Kelly, & McCreadie, 2000; Smith, Roberts, Brewer, & Pantelis, 1998). The NART is conceptually less sensitive to illness-related cognitive interferences during development and was not correlated with symptom level in the schizophrenia group. Nevertheless, in the bipolar group we found an association between NART IQ and both antipsychotic medication and symptoms as measured by GAF S at baseline.

Marked premorbid declines were found in the schizophrenia group. This is in line with previous findings, and with the concept of cognitive decline as illness-specific to schizophrenia (Meier et al., 2014) and a marker of conversion to psychosis (Woodberry et al., 2008). Using a retrospective measure of premorbid IQ, Ohi et al. (2017) found an intellectual decline in 70% of patients, which is at the same level as the estimated decline in our model. Studies have identified subgroups that vary in premorbid IQ and rate of decline, and identified associations with a clinical course (Vaskinn et al., 2020; Weickert et al., 2000). Although we did not investigate heterogeneity, these findings underscore the potential of early identification by estimating premorbid to post-onset IQ losses.

In the bipolar group premorbid IQ was less impaired, in accordance with findings of premorbid developmental deficits intermediate between controls and schizophrenia (Parellada, Gomez-Vallejo, Burdeus, & Arango, 2017), but discordant with evidence of high premorbid IQ as a risk factor (Smith et al., 2015). A small decline toward illness onset was only significant in comparison to the schizophrenia group. Given the small but significant associations with IQ for both medication and symptom level at baseline in the bipolar group, it is possible that the deficits at baseline are at least partly affected by illness-related factors and do not purely reflect developmental course. This would concur with the finding that premorbid deficits in bipolar disorder are only found in retrospective studies (Trotta et al., 2015).

All groups showed increases during follow-up, probably due to a combination of development and practice effects. The schizophrenia group showed slightly larger increases than controls during the follow-up period. However, they maintained lower levels than the control group for the duration of the follow-up period. These results dovetail with a previous study on premorbid to long-term IQ development in IQ-based schizophrenia subgroups (Van Winkel et al., 2007). They are also largely in line with findings of stability (Van Haren, Van Dam, & Stellato, 2019), but in contradiction with the idea of reduced gains over time (Hedman et al., 2013). Our findings do not support a hypothesis of neurodegeneration over the course of illness for bipolar disorder, at least not at the group level. Both patient groups recovered beyond their premorbid level and exhibited IQ scores that were within the average range. Still, the patient groups maintained a lag due to their lower premorbid levels, consistent with hypotheses of neurodevelopmental and cognitive risk factors (Melle, 2019; Radua et al., 2018).

To compare premorbid and current IQ, which were assessed with different measures, we used standardized IQ scores. However, to give an indication of actual IQ performance change from baseline to follow up, we also analyzed raw subtest scores from WASI. As expected, given the development of IQ in the general population (Hartshorne & Germine, 2015), increases were found in the Vocabulary subtest for all groups. Other subtests did not change significantly over time, indicating that IQ change was mainly driven by increased verbal skills. The absence of interactions implies similar developmental courses for all groups, also in IQ subfunctions.

As mentioned above, high symptom levels associated with acute onset phases could have interfered with test performance at baseline. For the bipolar group, small correlations between both current and premorbid IQ measures and symptom level were found. These associations were, however, not observed for the schizophrenia group, despite higher symptom levels and a marked decline in baseline IQ relative to premorbid estimates. Thus, these findings do not indicate that symptom severity is a primary explanation of the drop in IQ, although there might be group differences in the degree to which illness factors affect performance. We also investigated possible links with functioning. Van Winkel et al. (2007) found associations between functioning and both estimated premorbid IQ and IQ at follow-up, but not IQ at baseline, indicating that IQ measured close to illness onset may be less predictive of functional outcome. In the current study, however, we found no associations between IQ and functioning at either time-point.

Generally, antipsychotic medication has not been found to have a detrimental effect on cognition in FEP (Davidson et al., 2009), although these medications have been linked to poorer cognition in a middle-aged schizophrenia sample (Husa et al., 2017). Lithium, on the other hand, may have neuroprotective properties (Chuang & Manji, 2007; de Sousa et al., 2011; Nunes, Forlenza, & Gattaz, 2007) although long-term effects are not yet fully known (Samamé et al., 2014). We found a small dose-dependent effect of antiepileptic- and antipsychotic medication at baseline for the bipolar group. This result might be confounded by other illness-related factors, especially considering the observed correlation between symptom level and IQ at baseline. The absence of an association at follow-up implies that the medication effects are transient.

### Strengths and limitations

The study has several key strengths. There is a paucity of longitudinal studies including both schizophrenia and bipolar patients, as well as healthy controls. The inclusion of both patient groups makes a comparison of their intellectual trajectories possible, and the inclusion of a healthy control group enables comparison with normal developmental trajectories. Furthermore, the follow-up time of 10 years is to our knowledge the longest to date.

Some limitations also warrant mentioning. Development was modeled using two different measures of IQ (NART and WASI), raising the possibility of these measures tapping into different IQ concepts. Also, an assessment actually undertaken in the premorbid phase would have been preferable to an estimate of premorbid IQ at illness onset (Bora, 2014b). However, the yearly incidence of psychotic disorders is low, and sampling IQ from the ‘right’ individuals before onset poses a practical challenge. Measures like the NART are designed to tap earlier IQ levels and be minimally vulnerable to illness effects. In line with this, the NART predicts IQ acceptably except for extremes in the normal distribution (Nelson & Willison, 1991). The emphasis on verbal content could make the NART vulnerable to language difficulties. Excluding participants with dyslexia did however not change the effects found (see Online Supplementary Tables S10–S12).

Patients were recruited to follow-up from a translational research study using a time-consuming protocol at baseline. As often seen in recent similar longitudinal studies, many participants declined the invitation to come back (Leeson et al., 2011; Siegel et al., 2006; Van Winkel et al., 2007; Velthorst et al., 2017). The retention rate for the patients was therefore low and left us with a limited follow-up patient sample. Thus, to maximize power we used mixed models.

In follow-up dropout analyses, we found higher baseline IQ in the schizophrenia subsample that completed follow-up. Higher baseline IQ in schizophrenia participants who completed assessment compared to non-completers could indicate that we lost more of the severely ill participants to follow-up. However, we found no differences between them in GAF-S or GAF-F scores. Initial analyses on the follow-up sample, gave no indication of different developmental courses between a low- and high-performing subset. Notwithstanding these results, the slope from baseline to follow-up might be overestimated due to the difference in baseline- and follow-up samples, and the interaction effect should be interpreted with caution. This issue would however not affect estimated slopes from premorbid to baseline levels, as these are based on the entire baseline sample.

Finally, some evidence suggests that the Norwegian WASI overestimates IQ with between 0.5 and 1 standard deviation (Siqveland, Dalsbø, Harboe, & Leiknes, 2014). This may contribute to the high scores in our sample. The slopes can arguably be interpreted as indicators of development even assuming overestimated intercepts.

## Conclusion

IQ trajectories from premorbid levels through to 10-year follow up were investigated in schizophrenia participants, bipolar participants, and healthy controls. Starting from lower premorbid levels compared to healthy controls, the schizophrenia group showed significant and marked declines in IQ around illness onset, with a trend toward a milder drop in the bipolar group. During the follow-up period, the patient groups showed increases in their IQ levels, but reductions were maintained compared to the healthy controls. Still, these subsequent increases indicate a development of IQ in young adulthood in schizophrenia and bipolar disorder that mainly follows the trajectories of normal development.

## Supplementary material

For supplementary material accompanying this paper visit https://doi.org/10.1017/S0033291721004645. click here to view supplementary material
