# Supplementary material for: Course of intellectual functioning in schizophrenia and bipolar disorder: a 10-year follow-up study
Source: Psychol Med. 2022 Mar 8;53(6):2662–70. doi: 10.1017/S0033291721004645 (PMC10123835; doi:10.1017/S0033291721004645)
Supplement: Supplementary file 1 [file S0033291721004645sup001.docx]

**Supplementary**

***Table 6:*** ***Paired samples t-tests of difference between baseline and follow-up NART and WASI scores***

| Group | Test | t | df | p |
| --- | --- | --- | --- | --- |
| HC | NART | 1.55 | 90 | .125 |
|  | WASI | -4.09 | 89 | .000 |
| BD | NART | 0.51 | 24 | .612 |
|  | WASI | -2.67 | 30 | .012 |
| SZ | NART | -1.29 | 31 | .206 |
|  | WASI | -4.68 | 35 | .000 |

*SZ: schizophrenia group, BD: bipolar group, HC: healthy controls, NART: National Adult Reading Test, WASI: Wechsler Abbreviated Scale of Intelligence*

**Initial analyses, growth curve models, split by group**

***Table 7: Time modeled as linear effect***

| Parameter | Estimate | | | SE | | t | | sig. | | 95 % CI | |
| --- | --- | --- | --- | --- | --- | --- | --- | --- | --- | --- | --- |
|  | |  |  | |  | |  | | Lower | | Upper |
| HC  Intercept | | 113.37 | 0.60 | | 188.80 | | 0.000 | | 112.18 | | 114.56 |
| Time  AIC: 2314.25  BD | | 0.33 | 0.07 | | 4.90 | | 0.000 | | 0.20 | | 0.46 |
| Intercept | | 109.18 | 1.02 | | 106.83 | | 0.000 | | 106.99 | | 111.05 |
| Time | | 0.31 | 0.14 | | 2.17 | | 0.032 | | 0.003 | | 0.59 |
| AIC: 1262.87 | |  |  | |  | |  | |  | |  |
| SZ | |  |  | |  | |  | |  | |  |
| Intecept  Time  AIC: 2093.65 | | 102.64  -0.14 | 0.99  0.16 | | 103.82  -0.89 | | 0.000  0.377 | | 100.68  -0.46 | | 104.60  0.17 |

*SZ: schizophrenia group, BD: bipolar group, HC: healthy controls, AIC: Akaikes Information Criterion, Intercept: mean at baseline, Time: estimated increase per year with a linear effect of time.*

***Table 8: Time modeled as quadratic effect***

| Parameter | Estimate | SE | t | sig. | 95 % CI | |
| --- | --- | --- | --- | --- | --- | --- |
|  |  |  |  |  | Lower | Upper |
| HC  Intercept | 113.31 | 0.67 | 168.57 | 0.000 | 111.99 | 114.64 |
| Time | 0.41 | 0.40 | 1.02 | 0.311 | -0.39 | 1.20 |
| Time * Time  AIC: 2316.21  BD | -0.01 | 0.03 | -0.20 | 0.842 | -0.69 | 0.06 |
| Intercept | 110.56 | 1.15 | 96.40 | 0.000 | 108.29 | 112.84 |
| Time | -1.61 | 0.69 | -2.33 | 0.022 | -2.97 | -0.24 |
| Time * Time  AIC: 1257.10  SZ  Intercept | 0.15  106.65 | 0.05  1.04 | 2.84  102.42 | 0.006  0.000 | 0.05  104.60 | 0.26  108.71 |
| Time | -4.54 | 0.54 | -8.40 | 0.000 | -5.61 | -3.47 |
| Time * Time | -0.36 | 0,04 | 8.38 | 0.000 | 0.28 | 0.45 |
| AIC: 2034.62 |  |  |  |  |  |  |

*SZ: schizophrenia group, BD: bipolar group, HC: healthy controls, AIC: Akaikes Information Criterion, Intercept: mean at baseline, Time: estimated increase per year with a linear effect of time, Time*Time: estimated increase per year with a quadratic effect of time.*

***Table 9: Time modeled as separate slopes for Time 1 and Time 2***

| Parameter | Estimate | SE | t | sig. | 95 % CI | |
| --- | --- | --- | --- | --- | --- | --- |
|  |  |  |  |  | Lower | Upper |
| HC  Intercept | 113.31 | 0.54 | 210.94 | 0.000 | 112.25 | 114.37 |
| Time 1 | 0.40 | 0.35 | 1.14 | 0.260 | 1.10 | 1.10 |
| Time 2  AIC: 2285.59  BD | 0.30 | 0.06 | 4.37 | 0.000 | 0.43 | 0.43 |
| Intercept | 110.59 | 0.88 | 125.42 | 0.000 | 108.83 | 112.35 |
| Time 1 | -1.32 | 0.58 | -2.26 | 0.027 | -2.48 | -0.15 |
| Time 2  AIC: 1247.90  SZ  Intercept  Time 1  Time 2  AIC: 1995.51 | 0.54  107.61  -4.27  0.57 | 0.17  0.62  0.47  0.09 | 3.13  173.52  -9.11  6.65 | 0.004  0.000  0.000  0.000 | 0.19  106.39  -5.20  0.40 | 0.89  108.84  -3.35  0.74 |

*SZ: schizophrenia group, BD: bipolar group, HC: healthy controls, AIC: Akaikes Information Criterion, Intercept: mean at baseline, Time 1: estimated increase per year from premorbid to baseline, Time 2: estimated increase per year from baseline to follow-up*

**Multilevel analyses excluding participants with dyslexia**

| Parameter | Estimate | SE | t | sig. | 95 % CI | |
| --- | --- | --- | --- | --- | --- | --- |
|  |  |  |  |  | Lower | Upper |
| Intercept | 113.58 | 0.53 | 213.13 | 0.000 | 112.53 | 114.63 |
| Time 1 | 0.256 | 0.38 | 0.67 | 0.502 | -0.49 | 1.01 |
| Time 2 | 0.278 | 0.06 | 4.317 | 0.000 | 0.15 | 0.40 |
| Group | -5.60 | 0.89 | -6.30 | 0.000 | -7.35 | -3.85 |
| Time 1 x group | -4.03 | 0.62 | -6.47 | 0.000 | -5.25 | -2.80 |
| Time 2 x group | 0.35 | 0.12 | 2.84 | 0.005 | 0.11 | 0.59 |

***Table 10: Model parameters for comparison between schizophrenia and healthy controls, excluding dyslexia***

*SZ: schizophrenia group, HC: healthy controls, Intercept: estimated mean premorbid IQ for the schizophrenia group, Group: effect of healthy controls, Time 1: estimated increase per year from premorbid to baseline, Time 2: estimated increase per year from baseline to follow-up, Time 1 x group: interaction effect of Time 1 for the healthy controls, Time 2 x group: interaction effect of Time 2 for the healthy controls.*

***Table 11: Model parameters for comparison between bipolar and healthy controls, excluding dyslexia***

| Parameter | Estimate | SE | t | sig. | 95 % CI | |
| --- | --- | --- | --- | --- | --- | --- |
|  |  |  |  |  | Lower | Upper |
| Intercept | 113.58 | 0.64 | 177.31 | 0.000 | 112.32 | 114.85 |
| Time 1 | 0.25 | 0.37 | 0.69 | 0.493 | -0.47 | 0.98 |
| Time 2 | 0.29 | 0.07 | 3.85 | 0.000 | 0.14 | 0.43 |
| Group | -2.98 | 1.13 | -2.64 | 0.009 | -5.21 | -0.75 |
| Time 1 x group | -1.56 | 0.65 | -2.41 | 0.017 | -2.84 | -0.28 |
| Time 2 x group | 0.27 | 0.49 | 1.85 | 0.066 | -0.02 | 0.57 |

*BD: bipolar group, HC: healthy controls, Intercept: estimated premorbid IQ for the bipolar group, Group: effect of healthy controls, Time 1: estimated increase per year from premorbid to baseline, Time 2: estimated increase per year from baseline to follow-up, Time 1 x group: interaction effect of Time 1 for the healthy controls, Time 2 x group: interaction effect of Time 2 for the healthy controls.*

***Table 12: Model parameters for comparison between patient groups, excluding dyslexia***

| Parameter | Estimate | SE | t | sig. | 95 % CI | |
| --- | --- | --- | --- | --- | --- | --- |
|  |  |  |  |  | Lower | Upper |
| Intercept | 110.62 | 0.92 | 120.19 | 0.000 | 108.79 | 112.44 |
| Time 1 | -1.33 | 0.65 | -2.04 | 0.043 | -2.63 | -0.04 |
| Time 2 | 0.55 | 0.16 | 3.48 | 0.001 | 0.24 | 0.87 |
| Group | -2.64 | 1.25 | -2.11 | 0.037 | -5.11 | -0.16 |
| Time 1 x group | -2.46 | 0.89 | -2.78 | 0.006 | -4.21 | -0.71 |
| Time 2 x group | 0.08 | 0.22 | 0.35 | 0.731 | -0.36 | 0.51 |

*SZ: schizophrenia group, BD: bipolar group, Intercept: estimated premorbid IQ for the schizophrenia group, Group: effect of bipolar group, Time 1: estimated increase per year from premorbid to baseline, Time 2: estimated increase per year from baseline to follow-up, Time 1 x group: interaction effect of Time 1 for the bipolar group, Time 2 x group: interaction effect of Time 2 for the bipolar group.*
